# The hospital environment versus carriage: transmission pathways for third-generation cephalosporin-resistant bacteria in blood in neonates in a low-resource country healthcare setting

**DOI:** 10.1038/s41598-022-11626-6

**Published:** 2022-05-19

**Authors:** Dory Kovacs, Vitus Silago, Delfina R. Msanga, Stephen E. Mshana, Jeremiah Seni, Katarina Oravcova, Louise Matthews

**Affiliations:** 1 Boyd Orr Centre for Population and Ecosystem Health and Institute of Biodiversity, Animal health and Comparative Medicine, University of Glasgow, Glasgow, UK; 2 Department of Microbiology and Immunology, Catholic University of Health and Allied Sciences, Mwanza, Tanzania; 3 Department of Paediatrics and Child Health, Catholic University of Health and Allied Sciences, Mwanza, Tanzania

**Keywords:** Bacteriology, Infectious diseases, Neonatal sepsis

## Abstract

Neonatal bloodstream infections (BSI) can lead to sepsis, with high morbidity and mortality, particularly in low-income settings. The high prevalence of third-generation cephalosporin-resistant organisms (3GC-RO) complicates the management of BSI. Whether BSI is linked to carriage of 3GC-RO, or to acquisition from the hospital environment is important for infection prevention and control, but the relationship remains unclear, especially in low-income settings. At a tertiary hospital in Mwanza, Tanzania, we screened neonatal blood and rectal samples from 200 neonates, and 400 (hospital) environmental samples. We used logistic regression to identify risk factors, and Kolmogorov–Smirnov tests and randomisation analyses to compare distributions of species and resistance patterns to assess potential routes of transmission. We found that BSIs caused by 3GC-RO were frequent (of 59 cases of BSI, 55 were caused by 3GC-RO), as was carriage of 3GC-RO, particularly *Escherichia coli*, *Klebsiella pneumoniae*, and *Acinetobacter* species. In the 28 infants with both a carriage and blood isolate, there were more (4 of 28) isolate pairs of the same species and susceptibility profile than expected by chance (*p* < 0.05), but most pairs were discordant (24 of 28). Logistic regression models found no association between BSI and carriage with either 3GC-RO or only 3GC-R *K. pneumoniae*. These analyses suggest that carriage of 3GC-RO is not a major driver of BSI caused by 3GC-RO in this setting. Comparison with environmental isolates showed very similar distributions of species and resistance patterns in the carriage, BSI, and the environment. These similar distributions, a high frequency of *Acinetobacter* spp. isolations, the lack of strong association between carriage and BSI, together with the high proportion of 3GC-RO in BSI all suggest that these neonates acquire multidrug-resistant carriage and blood isolates directly from the hospital environment.

## Introduction

Neonatal infections, such as blood stream infections (BSI) are known to account for a significant proportion of deaths occurring in the first 28 days of life^1^. Premature neonates and those with low birthweight are most vulnerable to BSI, and consequently sepsis, due to inadequate immune barriers and responses^2^. The most common bacteria causing BSI in low- or middle-income countries (LMICs) are different to those in high-income countries (HIC)^3^. In HICs, the most isolated species are Group B *Streptococcus*, *Escherichia coli,* and coagulase negative staphylococci^3,4^, while *S. aureus, E. coli*, *Klebsiella pneumoniae*, and *Acinetobacter* spp., are the most common causes of neonatal sepsis in LMICs^2,3,5,6^.

The diagnosis of neonatal sepsis is often delayed due to its subtle and non-specific initial signs^7^. Risk factors include prematurity, low birthweight, premature rupture of membranes, and maternal infections at the time of delivery^8^. Early-onset neonatal sepsis, typically defined as presenting within 72 h of birth, is often associated with organisms from the maternal flora, known as vertical transmission^7,9^. In contrast, late onset neonatal sepsis (> 72 h) is thought to be caused by pathogens acquired from elsewhere, usually the hospital environment^7,10^. However, this view is shifting, as difficult or unhygienic births may result in neonatal infections resulting from environmental, rather than maternal, pathogens acquired < 72 h^6^.

Since neonatal BSI is a life-threatening condition that can rapidly progress to septic shock, physicians prescribe empirical treatment before microbiology results are available. The World Health Organization’s (WHO) guidelines recommend intravenous ampicillin and gentamicin as prophylaxis in neonates with documented risk factors (such as maternal fever (> 38 °C) before or during delivery) for at least two days, and continuing for at least ten days in cases of suspected sepsis^11^. However, these guidelines were developed with no comprehensive surveillance data from neonatal units in LMIC settings^12^.

Antimicrobial resistance (AMR) jeopardises the effective treatment of neonatal BSI by Gram-negative organisms. The emergence and spread of extended-spectrum β-lactamases (ESBLs) pose an urgent public health risk^13^, as they confer resistance to third-generation cephalosporins and monobactams, while the plasmids encoding ESBLs often carry additional genes that confer resistance to aminoglycosides, tetracycline, and fluoroquinolones^14,15^. The WHO classifies ESBL-producing Enterobacteriaceae as critical priority I pathogens, requiring urgent research and development of new therapies^16^.

Third-generation cephalosporin-resistant organisms (3GC-RO) are highly prevalent in East Africa^17–20^. Gentamicin resistance is also a well-recognised issue here^21^, and has been reported to be as high as 84% in septic neonates^22^, with significantly more frequent resistance in 3GC-RO^18^.

Therefore, there is an urgent need to map the sources and transmission pathways of 3GC-RO in healthcare settings. *K. pneumoniae* has been shown to colonise the gastrointestinal tract of preterm infants and their immediate hospital environment, with frequent exchange between the two sources^23^. Whilst previous studies have shown an association between colonisation and BSI^24^, there is little data available for LMIC settings, and the extent to which the hospital environment versus intestinal colonisation is the source for blood stream infections with 3GC-RO remains unclear. Improved understanding of these sources and pathways would help direct infection prevention and control (IPC) measures.

The aims of this study were to quantify the burden of 3GC-RO in neonates; to identify risk factors associated with both 3GC-RO rectal carriage and BSI; and to identify potential transmission pathways by comparing the distributions of organisms and their multi-drug resistance patterns between the neonates and their environment and examining the concordance between carriage and blood isolates.

## Methods

### Study setting and population

Bugando Medical Center (BMC) is a 950-bed tertiary hospital in Mwanza, Tanzania. At the time of the study, there were 60 cots in the neonatal wards, of which 15 were on the neonatal intensive care unit (NICU). There were four functional hand washing sinks in total (two of these were in the NICU). Alcohol rubs were frequently used by paediatricians and infrequently by nurses. At any given time, there were four paediatricians and seven neonatal nurses on shift, of whom four worked in the NICU.

Patient and microbiology data were obtained from a previously described study^25^. In brief, isolates and data were collected over a four-month period. We enrolled a total of 200 neonates (under 28 days of age) born at or admitted to BMC between January and April 2019. Neonates with signs and symptoms of infections (as defined by The WHO Young Infants Study Group^26^) and their mothers/guardians were recruited serially until the sample size was reached. Variables collected were as previously described^25^. Informed parental consent was sought for participation, and a questionnaire was completed, including questions on maternal health, for example antibiotic use and fever during pregnancy or labour. Information on neonatal health, including antibiotic treatment, was obtained from the patients’ medical records. Data were stripped of all personal identifiable information prior to analyses, and all methods were carried out in accordance with the relevant guidelines and regulations set by the Declaration of Helsinki.

### Sample collection and culture

A blood sample and a rectal swab were obtained from all neonates (n = 200) at the time of enrolment. This was as soon as possible after birth or admission (usually within hours). A total of 143 neonates were enrolled < 48 h of birth. The median duration of stay before sampling was 1 (IQR 1–2) days. At the time of rectal sampling, maternal hands and neonatal cots were also swabbed—these were considered to represent organisms in the hospital environment. Blood samples were cultured for all bacterial growth, while only 3GC-RO were cultured for from rectal and environmental swabs using cefotaxime-supplemented MacConkey agar. Laboratory procedures, including bacterial culture and identification, were previously described^25^. For blood stream samples, any bacterial growth indicated BSI; we excluded non-3GC-R isolates from further analysis.

### Antimicrobial susceptibility testing

Antimicrobial susceptibility testing (AST) of isolates was carried out by disc diffusion method using the antibiotics listed in Table 1 following the Clinical and Laboratory Standard Institute guidelines (2018 version,^27^). As rectal and environmental swabs were selectively grown on cefotaxime-supplemented MacConkey agar, only blood isolates were tested for cefotaxime resistance.

**Table 1:** Antimicrobials used for AST by disc diffusion for neonatal and environmental isolates.

Antimicrobial	Class
Amikacin	Aminoglycoside
Cefoxitin	Second-generation cephalosporin
Cefotaxime (blood only)	Third-generation cephalosporin
Ciprofloxacin	Fluoroquinolone
Gentamicin	Aminoglycoside
Meropenem	Carbapenem
Tetracycline	Tetracycline
Trimethoprim–sulfamethoxazole	Folic acid inhibitors/sulfonamides

### Data analysis

Neonatal information obtained from the questionnaires, patient records and microbiological tests were anonymised, aggregated, and analysed using R version 3.5.3 (2019-03-11).

#### Comparison of species distributions and resistance patterns

Distributions of species abundance were compared using a bootstrap Kolmogorov–Smirnov (KS) test. The KS test statistic was the maximum absolute difference in the cumulative distributions of the species distributions being compared. A total of 10,000 test samples, each with the same number of observations as the test distribution, were computed along with the test statistic to generate the expected distribution of the test statistic. In addition, frequencies of resistance between isolate sources were compared for the most common species isolated. The proportion of isolates resistant to a given antibiotic was compared between isolate sources using *prop.test()* in R. When multiple comparisons were made, the Bonferroni correction was applied.

#### Randomisation analysis of organisms and resistance patterns

Random resampling of the observed blood and carriage isolates was used to assess whether the observed number of matches in a neonate by organisms and resistance patterns across BSI and rectal carriage isolates were greater than expected by chance. For this, 28 (the number of neonates that had an organism isolated both from their blood and a 3GC-RO isolate from their rectal swab) organisms were randomly sampled from the set of neonatal blood isolates and 28 from the set of carriage isolates and the number of concordant pairs counted. This was done 100,000 times to obtain a distribution of the expected number of concordant pairs under a null hypothesis of no relationship between carriage and blood isolates. To assess robustness, the process of random re-sampling was repeated restricting the rectal organisms to only those found in cases of BSI.

#### Risk factor analyses for infection and carriage

We used two sets of logistic regression models to investigate risk factors associated with (1) neonatal carriage of 3GC-RO and (2) bloodstream infections. The outcome variables were infection/carriage with any 3GC-RO or just 3GC-R *K. pneumoniae*, the most prevalent 3GC-RO in carriage and blood. We included covariates that have been previously associated with infection and carriage or if a suspected biological link with the outcome existed (Table 2). Stepwise backwards model selection was then performed to identify the model with the lowest Akaike Information Criterion (AIC). Maternal antibiotic use and fever during pregnancy were found to be strongly associated (χ^2^ = 49.2, *p* < 0.001), and so the models were run including one or the other, to find the model with the lowest AIC. Residuals were assessed using standardised residuals generated using the DHARMA package^28^ in R and assessed for normality and dispersion.

**Table 2:** Covariates used in the two sets of logistic regression models.

Covariate	Levels (n = 200)	Carriage	Infection
Maternal antibiotics	Yes (n = 64)/No	✓	✓
Maternal fever during pregnancy	Yes (n = 95)/No	✓	✓
Neonatal antibiotics	Yes (n = 175)/No	✓	✓
Enrolment time	< 48 h (148)/> 48 h	✓	✓
Carriage of 3GC-RO	Yes (n = 86)/No	NA	✓
Presence of 3GC-RO on cot	Yes (n = 64)/No	✓	✓
Intravenous line used	Yes (n = 163)/No	✓	✓
Premature rupture of membranes	Yes (n = 17)/No	✓	✓
Meconium-stained liquor	Yes (n = 33)/No	✓	✓
Sex	Male (n = 105) versus female	✓	✓
Place of delivery	Home (n = 14) versus hospital	✓	✓
Mode of delivery	Vaginal (n = 120) versus Caesarean section	✓	✓
Ward	Neonatology (n = 116) versus NICU	✓	✓
Birthweight	Continuous (in kg)	✓	✓

*3GC-RO* third-generation cephalosporin-resistant organism, *NA* not applicable.

### Ethics approval and consent to participate

Protocols and procedures in this study were approved by Code of Conduct for Research Ethics of the Sokoine University of Agriculture with certificate number: SUA/CVMBS/R.1/2018/8 and ethically cleared by the joint CUHAS/ BMC Research Ethics and Review Committee (CREC) with certificate number: CREC/298/2018. All participants signed an informed consent before enrolment, except for participants aged < 18 years, where informed consent of participation was provided by their parents/guardians. Detailed microbiological reports of clinical specimens were shared in a timely manner with attending doctors in respective units for proper neonates’ management.

## Results

### Distributions of organisms by source

**Figure 1:**
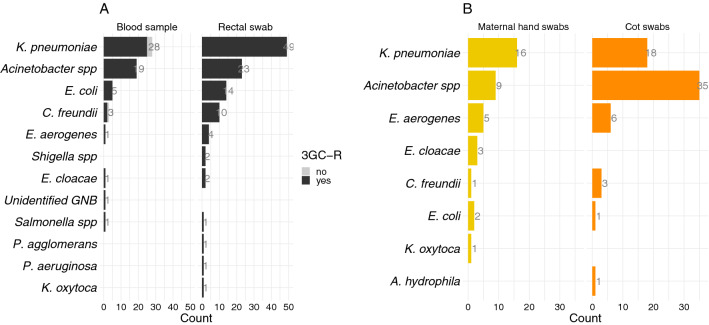
Gram-negative bacteria isolated from blood, and rectal, maternal hand and cot swabs, (**A**) Gram-negative organisms isolated from blood samples and rectal swabs. Dark grey shows 3GC-RO, light grey 3GC-susceptible. (**B**) Gram-negative organisms isolated from cot (orange) and maternal hand swabs (yellow), all 3GC-R.

There were 59 Gram-negative organisms isolated from blood (from 59 neonates), and 108 from rectal swabs (from 86 neonates). There were 22 neonates who had two different 3GC-R organisms isolated from their rectal swab. There were 101 environmental 3GC-RO isolates: 37 from maternal hands and 64 from cots. 3GC-R *K. pneumoniae* was most prevalent in blood samples (n = 28), rectal swabs (n = 49), and maternal hand swabs (n = 16), and second most prevalent in cot swabs (n = 18). *Acinetobacter* spp. was most common in cot swabs (n = 35), and second most common in all other types of samples and swabs (Fig. 1A and B). Pairwise Kolmogorov–Smirnov tests showed no significant difference between the distributions of organisms by source (blood samples, rectal swabs, neonatal cot, maternal hands; Table 3).

**Table 3:** Comparison of species distributions of organisms isolated from blood, rectal, cot and maternal hands, using a Kolmogorov–Smirnov tests.

Test	*P* value
Distribution of organisms isolated from blood versus rectal swabs	0.3735
Distribution of organisms isolated from cot versus maternal hand swabs	0.8468
Distribution of organisms isolated from blood versus maternal hand swabs	1
Distribution of organisms isolated from rectal versus maternal hand swabs	0.4982
Distribution of organisms isolated from rectal versus cot swabs	0.474
Distribution of organisms isolated from blood versus cot swabs	0.7542
Distribution of organisms isolated from blood in hospital versus home deliveries	0.8495
Distribution of organisms isolated from rectal swabs in hospital versus home deliveries	0.0163

As eight tests were conducted, the threshold for significance after the Bonferroni correction was 0.05/8 = 0.00625.

### Resistance patterns in neonatal infection and carriage isolates

**Figure 2:**
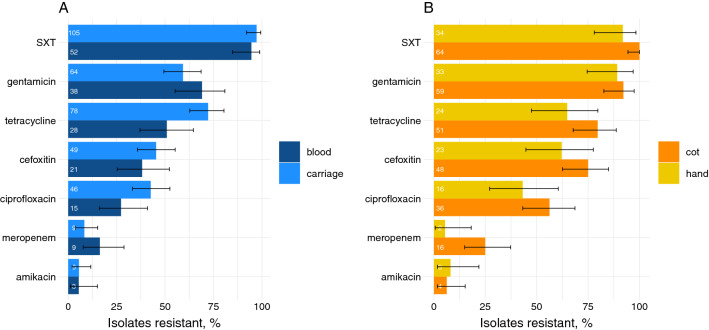
Frequencies of resistances in Gram-negative bacteria isolated from blood, and rectal, maternal hand and cot swabs. (**A**) Frequencies of antimicrobial resistances in blood (dark blue) and carriage (light blue), 3GC-RO only. (**B**) Frequencies of antimicrobial resistances in cot (orange) and maternal hand swabs (yellow), all 3GC-RO. Error bars show 95% confidence intervals. *SXT* trimethoprim–sulfamethoxazole.

The most prescribed neonatal antibiotics were gentamicin and ampicillin. Resistance to gentamicin was frequent (> 60%) in 3GC-R blood and rectal isolates (Fig. 2A). Resistance to other antibiotics was also frequent, including trimethoprim–sulfamethoxazole (> 90% isolates resistant) and tetracycline (> 50% of isolates resistant). Amikacin resistance was rare (< 1%) in all isolate types. Frequencies of antimicrobial resistances in 3GC-R *K. pneumoniae* isolates (Fig. S3) were similar in blood, carriage, and environmental isolates apart from tetracycline. Tetracycline resistance was significantly more frequent in carriage compared to blood isolates (*p* = 0.012).

### Resistance patterns in 3GC-R organisms in environmental swabs

Multi-drug resistance, commonly defined as resistance to three or more classes of antimicrobials, was frequently detected in 3GC-RO isolated from neonatal cots and maternal hand swabs. Resistance to trimethoprim–sulfamethoxazole and gentamicin were also frequently detected (> 85% of isolates, Fig. 2B). Most isolates were susceptible to amikacin (> 90%).

### Association between neonatal carriage and blood isolates

**Figure 3:**
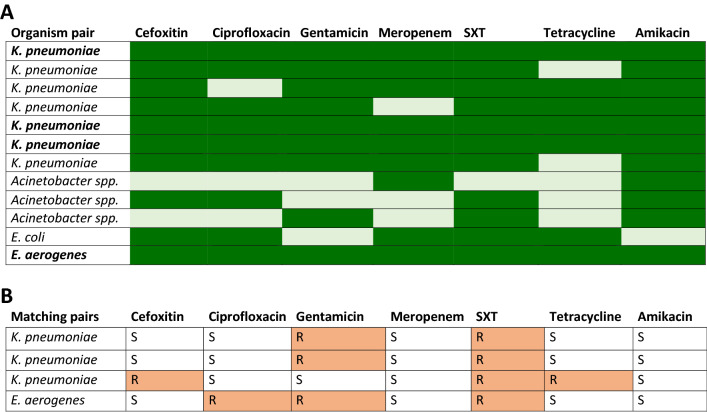
A comparison of resistance profiles of 3GC-R Gram-negative organisms isolated from blood and rectal swabs. (**A**) Twelve neonates had the same organism in their blood and rectum. Dark green indicates a match in resistance/susceptibility between the two isolates, light green represents a mismatch. Four pairs had identical resistance profiles (bold). (**B**) Breakdown of antibiotic susceptibility profiles of the four organisms with identical resistance patterns isolated from blood and rectum. *SXT* trimethoprim–sulfamethoxazole, *S* susceptible, *R* resistant.

There were 28 neonates from whom both a blood and carriage isolate were obtained. Of the 28, 16 neonates had distinct 3GC-RO species in their rectal swab and blood isolates and 12 had the same 3GC-RO species (Fig. 3A).

**Figure 4:**
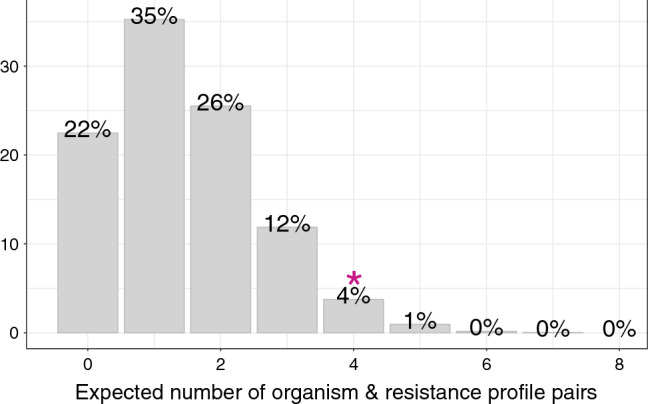
Expected number of matching organism and resistance profile pairs in neonatal blood and carriage isolates.

Four of these pairs shared identical resistance profiles against cefotaxime, ciprofloxacin, gentamicin, meropenem, trimethoprim–sulfamethoxazole, tetracycline, and amikacin (Fig. 3B), with most mismatches in antimicrobial resistance patterns seen in *Acinetobacter* spp. (Fig. 3A). Under the null hypothesis of no association between carriage and blood isolates, the probability of detecting four organisms with matching resistance patterns was < 0.05 (Fig. 4).

### Risk factors associated with neonatal BSI and carriage of 3GC-R organisms

#### BSI due to 3GC-RO

For BSI caused by any 3GC-RO, the model that included maternal fever during pregnancy was found to be a better fit to our data (final model AIC = 224.77), compared with maternal antimicrobial use (final model AIC = 226.79). Similarly, for BSI caused by 3GC-R *K. pneumoniae*, the model including maternal fever during pregnancy was found to be a better fit to our data (final model AIC = 143.90), compared with maternal antimicrobial use (final model AIC = 144.81).

The final models were similar for BSI caused by any 3GC-RO and BSI caused by 3GC-R *K. pneumoniae* (Table 4). BSI caused by any 3GC-RO was positively associated with cot 3GC-RO contamination (OR 1.99, 95% CI 1.00–3.94), enrolment (sampling) time > 48 h (OR 2.32, 95% CI 1.14–4.69), and fever during pregnancy (OR 2.08, 95% CI 1.04–4.22). Hospital delivery was negatively associated with both BSI caused by any 3GC-RO (OR 0.30, 95% CI 0.09–1.00). BSI caused by 3GC-R *K. pneumoniae* positively associated with fever during pregnancy (OR 3.50, 95% CI 1.32–10.7), and negatively associated with hospital deliveries (OR 0.17, 95% CI 0.04–0.76).

**Table 4:** Final logistic regression models for neonatal BSI caused by and carriage of all 3GC-RO and 3GC-R *K. pneumoniae* only.

Covariate	Any 3GC-RO	3GC-R *K. pneumoniae*
	OR (95% CI)	OR (95% CI)
**Bloodstream infection**		
Intercept	0.48 (0.14–1.54)	**0.21 (0.05**–**0.71)**
Hospital delivery	**0.30 (0.09**–**1.00)**	**0.17 (0.04**–**0.76)**
Cot 3GC-RO colonisation	**1.99 (1.00**–**3.94)**	NA
Enrolment time (> 48 h)	**2.32 (1.14**–**4.69)**	2.34 (0.93–5.83)
Fever during pregnancy	**2.08 (1.04**–**4.22)**	**3.50 (1.32**–**10.7)**
Premature rupture of membranes	NA	3.40 (0.94–11.1)
**Carriage**		
Intercept	0.91 (0.33–2.65)	**0.23 (0.07**–**0.65)**
Neonatal antibiotics	**0.29 (0.11**–**0.73)**	0.43 (0.17–1.12)
Cot 3GC-RO colonisation	**2.52 (1.32**–**4.89)**	NA
Cot 3GC-R *K. pneumoniae* colonisation	NA	2.48 (0.81–7.30)
Female sex	**2.25 (1.23**–**4.15)**	**2.21 (1.07**–**4.65)**
Neonatology ward	1.67 (0.90–3.14)	1.83 (0.89–3.94)
Meconium-stained liquor	0.48 (0.20–1.12)	NA

Odds ratios shown with 95% confidence interval.

Significant associations are shown in bold.

*PROM* premature rupture of membranes, *3GC-RO* third-generation cephalosporin resistant organism, *NA* not applicable.

#### Carriage of 3GC-RO

The final models were similar for carriage of any 3GC-RO and 3GC-R *K. pneumoniae* (Table 4)*.* Cot 3GC-RO colonisation (OR 2.52, 95% CI 1.32–4.89) and female sex (OR 2.25, 95% CI 1.23–4.15) were positively associated with carriage of 3GC-RO, while neonatal antibiotic use was negatively associated (OR 0.29, 95% CI 0.11–0.73) with carriage of 3GC-RO. Female sex was also positively associated (OR 2.21, 95% CI 1.07–4.65) with carriage of 3GC-R *K. pneumoniae.*

### Organisms isolated from neonates born at home versus in hospital

Although place of delivery was a significant risk factor in neonatal BSI, Pairwise Kolmogorov–Smirnov tests showed that there was no significant difference between the distribution of blood isolates or carriage isolates from neonates born at home versus in hospitals (Fig. S2, Table 3).

## Discussion

To address the question of the relationship between blood stream infection, colonisation, and the hospital environment, we collected blood, rectal, and environmental (maternal hand and cot) samples from neonates admitted to BMC, a tertiary hospital in Mwanza, Tanzania, and quantified the prevalence and phenotypic resistance patterns of 3GC-R organisms. Non-invasive maternal hand swabs were considered to reflect transient microflora or organisms present in the environment, rather than maternal colonisation. We investigated risk factors associated with neonatal colonisation with and infection caused by 3GC-RO.

The most common bacteria causing BSI in LMICs are thought to be different from those in high-income countries^3^. In this study, *K. pneumoniae* and *Acinetobacter* spp. were the most prevalent 3GC-R organisms in blood and carriage isolates. Around a quarter of environmental samples were also positive for 3GC-RO, with 3GC-R *Acinetobacter* spp. and *K. pneumoniae* being the most frequently isolated organisms. This is not uncommon as *Acinetobacter* spp. has been often isolated from skin of healthy individuals^29^. We found no difference between the distribution of organisms across sampling sites. Studies have shown that *S. aureus, E. coli, K. pneumoniae*, and *Acinetobacter* spp. are the most common causes of sepsis in LMICs^2,3^ and our study confirms the predominance of *K. pneumoniae, Acinetobacter* spp., and *E. coli* in neonatal BSI, with the majority being 3GC-R. These results were also consistent with previous studies set in Tanzania^30,31^. Gram-negative organisms in the blood samples were significantly (*p* < 0.001) more prevalent than Gram-positive bacteria (59/69 vs 10/69).

Most neonates enrolled in this study were premature and/or had low birthweight (Fig. S1), putting them at high risk of BSI. Previous studies have linked a number of risk factors to neonatal BSIs, including fever during pregnancy or labour^32,33^, prematurity, male sex, prolonged rupture of membranes^34^, and colonisation of babies with microorganisms such as *K. pneumoniae*^35^. Here, fever in pregnancy and home deliveries were associated with an increased risk of BSI with any 3GC-R organism and with just 3GC-R *K. pneumoniae*. Delivery at home was also associated with higher neonatal mortality in a study set in Ethiopia^36^. However, this finding could reflect the health status of infants being admitted rather than the difference between home and hospital deliveries per se*.* Sampling neonates > 48 h after admission was also associated with increased risk of BSI caused by any 3GC-RO.

The risk of gastrointestinal carriage of 3GC-RO was positively associated with female sex and cot 3GC-RO colonisation, and negatively associated with neonatal antibiotic use. Carriage of such organisms has been reported to cause subsequent BSI^24,35,37,38^ but was not found to be a risk factor in this study.

There were four neonates with the same organism and resistance pattern in their blood and carriage isolates, which is more than expected by chance alone (*p* < 0.05). However, the more frequent occurrence of discordant resistant organism pairs in blood and carriage isolates suggests that carriage and BSI may be acquired independently. The potential link between rectal carriage and BSI could be further examined using whole genome sequence analysis, which provides a greater degree of discrimination between isolates. This could increase the number of discordant pairs and add support to the conclusion that colonisation is not the major source of BSI in this setting.

The current guidelines recommending the use of ampicillin and gentamicin in suspected neonatal sepsis are primarily based on clinical trials conducted in high-income settings^3^ where Gram-positive bacteria are the primary cause of neonatal BSI. In contrast, Fuchs et al*.* (2016) report that approximately 70% of pathogens isolated from paediatric sepsis in Tanzania are Gram-negative species, with *K. pneumoniae* (naturally ampicillin-resistant) and *E. coli* being the most prevalent. In addition, the common occurrence of 3GC-RO with co-resistance to gentamicin in the present and previous studies^14,15^ indicates that empirical treatment with ampicillin and gentamicin may not always be appropriate in these settings and may need to be altered after AST profiles become available for the isolates. In our setting, > 60% of 3GC-R organisms isolated from blood and rectal swabs and > 85% from cot and hand swabs were gentamicin resistant. The majority of 3GC-R *K. pneumoniae* isolated from blood samples were gentamicin resistant, as well as 58% of *Acinetobacter* spp. and 40% of *E. coli*. Among the most frequently isolated 3GC-RO in rectal swabs, *Acinetobacter* spp. was most frequently resistant to gentamicin, followed by *K. pneumoniae* and *E. coli*.

As previously reported, over 25% of neonates in this study had 3GC-RO in their blood^19^ and most blood isolates were 3GC-RO. Other than acquisition from the hospital environment, a possible explanation for the high proportion of 3GC-R organisms in blood is treatment with antibiotics clearing the non-3GC-R organisms.

Our study shows similar distributions of organisms and resistance patterns between isolates obtained from blood, carriage, and the environment, with *Acinetobacter* spp. frequently identified in all these isolates. Although carriage and blood isolate pairs were mostly discordant, there was more similarity between carriage and blood isolates than expected by chance. These observations support some association between carriage and BSI, which could be strengthened by sampling, AST, and genomic characterisation of multiple carriage isolates. However, frequent discordant pairs of isolates also support the hypothesis that carriage and blood isolates can be acquired independently, and more hospital environmental surveillance is needed in this respect. In addition, the frequent occurrence of *Acinetobacter* spp. suggests that the hospital environment may be a common source for both the carriage 3GC-R organisms and the blood organisms. Moreover, the high prevalence of 3GC-RO among the blood isolates suggests that the hospital environment may be a reservoir for 3GC-RO. Whilst previous studies have identified an association between colonisation and infection with concordant isolates, there is limited data on this relationship in low-income settings^24^.

Early-onset neonatal sepsis is generally defined as bacteraemia or bacterial meningitis that presents ≤ 72 h of birth, and is usually associated with organism acquired from the maternal flora^7,39^. Most neonates were sampled within 48 h of birth in this study, with high proportions of neonatal BSI caused by 3GC-RO. This either reflects the frequent carriage of 3GC-RO in mothers or indicates that these organisms are acquired from the hospital environment immediately after birth. The high frequency of 3GC-R *Acinetobacter* spp., organisms commonly associated with the hospital environment^29^, supports the latter. This is also concordant with a recent review by Sands et al.^6^, who highlight the potential role of environmental pathogens in early-onset neonatal sepsis. Nevertheless, as this study could not pinpoint the time of sepsis onset, the roles of the environment and gastrointestinal colonisation need further characterisation. In addition, we compared isolates from different sources based on phenotypic, rather than genetic data. The latter would be needed to confirm whether the same organisms are found in carriage, infection, and the hospital environment.

This study demonstrates an alarmingly high resistance to the commonly used antibiotics at neonatal wards at BMC, a major tertiary hospital in Tanzania. Given the availability of laboratory infrastructures and capacity at this institution, our findings highlight the need to provide antibiotic therapies guided by local epidemiology including antimicrobial susceptibility testing. Moreover, this study suggests that the major route of acquisition of both carriage and bloodstream isolates may be the hospital environment, rather than BSI arising from carriage organisms, indicating that improved IPC focused on the healthcare setting sources could be beneficial. These findings have implications for IPC and antibiotic treatment, indicating that IPC may need to focus on reducing hospital environmental reservoir; and that susceptibility profiling to inform antibiotic therapy might operate best at a group rather than individual level.

## Supplementary Information

Supplementary Figures.

## Data Availability

The datasets generated for the current study are available by reasonable request to the corresponding author.

## Abbreviations

3GC-R: Third-generation cephalosporin-resistant
3GC-RO: Third-generation cephalosporin-resistant organism
AIC: Akaike Information Criterion
AST: Antimicrobial susceptibility testing
BSI: Bloodstream infection
ESBL: Extended-spectrum beta-lactamase
IPC: Infection prevention and control
KS: Kolmogorov–Smirnov
LMIC: Low- and middle-income country
NICU: Neonatal intensive care unit
SXT: Trimethoprim–sulfamethoxazole
