# Supplementary material for: The hospital environment versus carriage: transmission pathways for third-generation cephalosporin-resistant bacteria in blood in neonates in a low-resource country healthcare setting
Source: Sci Rep. 2022 May 19;12:8347. doi: 10.1038/s41598-022-11626-6 (PMC9120020; doi:10.1038/s41598-022-11626-6)
Supplement: Supplementary file 1 — Supplementary Figures. [file 41598_2022_11626_MOESM1_ESM.pdf]

The hospital environment versus carriage: transmission pathways for third-generation cephalosporin-resistant bacteria in blood in neonates in a low-resource country healthcare setting

Supplementary material

Neonatal characteristics

The mean age of neonates on the NICU was 1.6 (SD 0.83) days at the time of enrolment, and 2.6 (SD 4) days in neonatology. The average gestational age was 31.8 weeks, 7 weeks fewer than full term (39 weeks). Mean birthweight was 1.7kg (SD 0.41), and all neonates had low (<2.50kg) or very low (<1.50kg) birthweight (Figure S1). Neonates considered having an infection or at risk of infection by clinicians received ampicillin (n=170) and gentamicin (n=166). Three neonates received a third-generation cephalosporin (ceftriaxone), while 25 (12.5%) were prescribed no antibiotics. There were 18 deaths, 12 of which occurred in NICU.

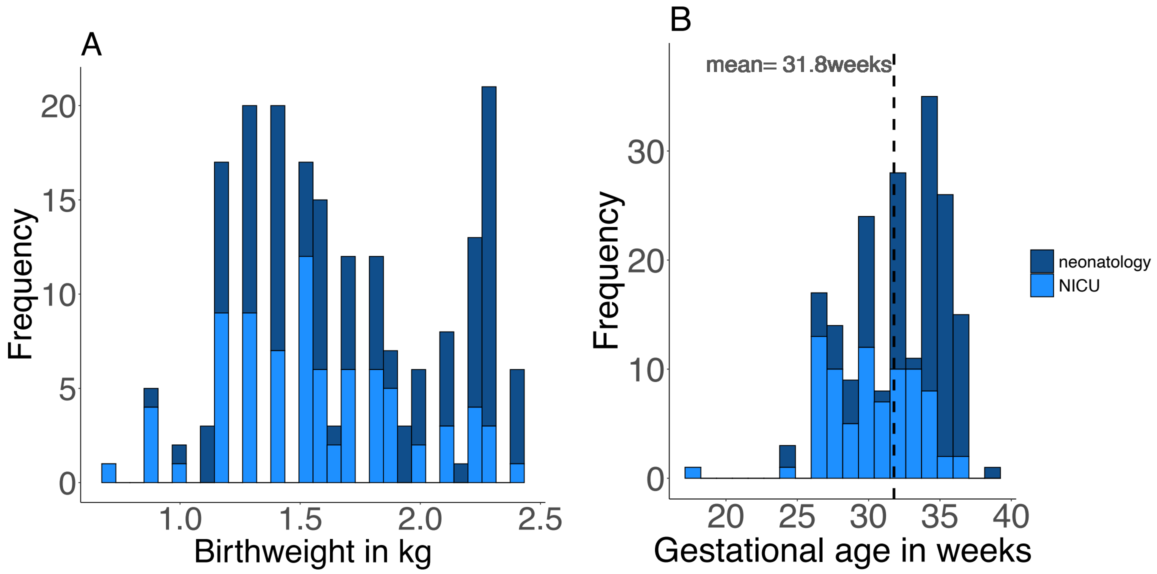

Figure S1 Neonatal characteristics. A) Birthweight, B) Gestational age. Dark blue shows neonatology and light blue shows NICU patients.

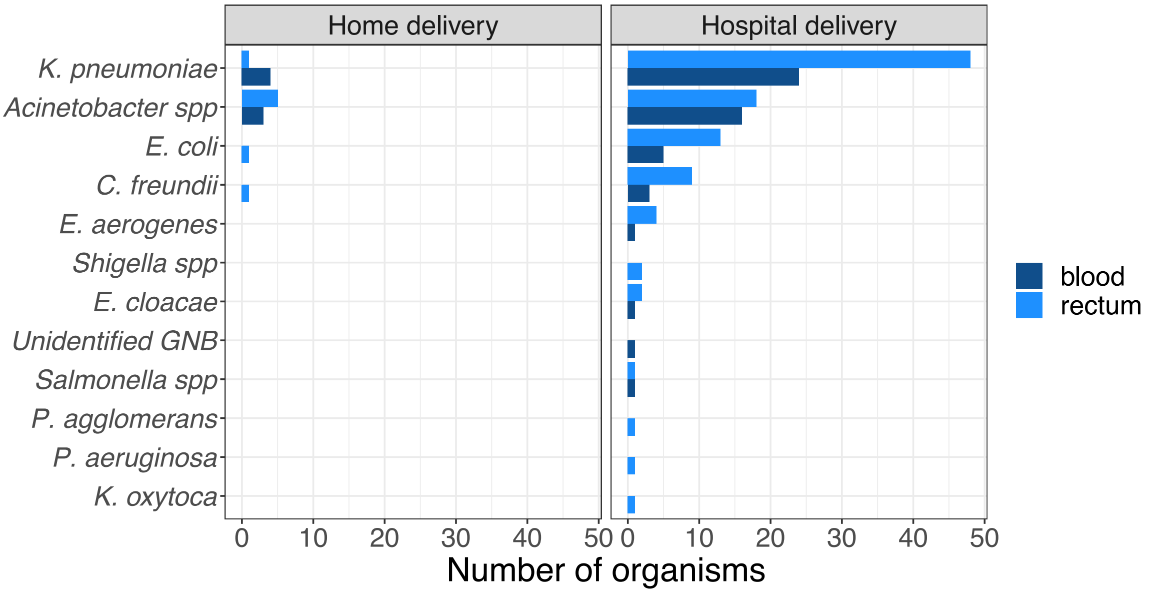

Figure S2 Number of organisms by delivery place (home vs hospital) in blood (dark blue) and rectal (light blue) samples.

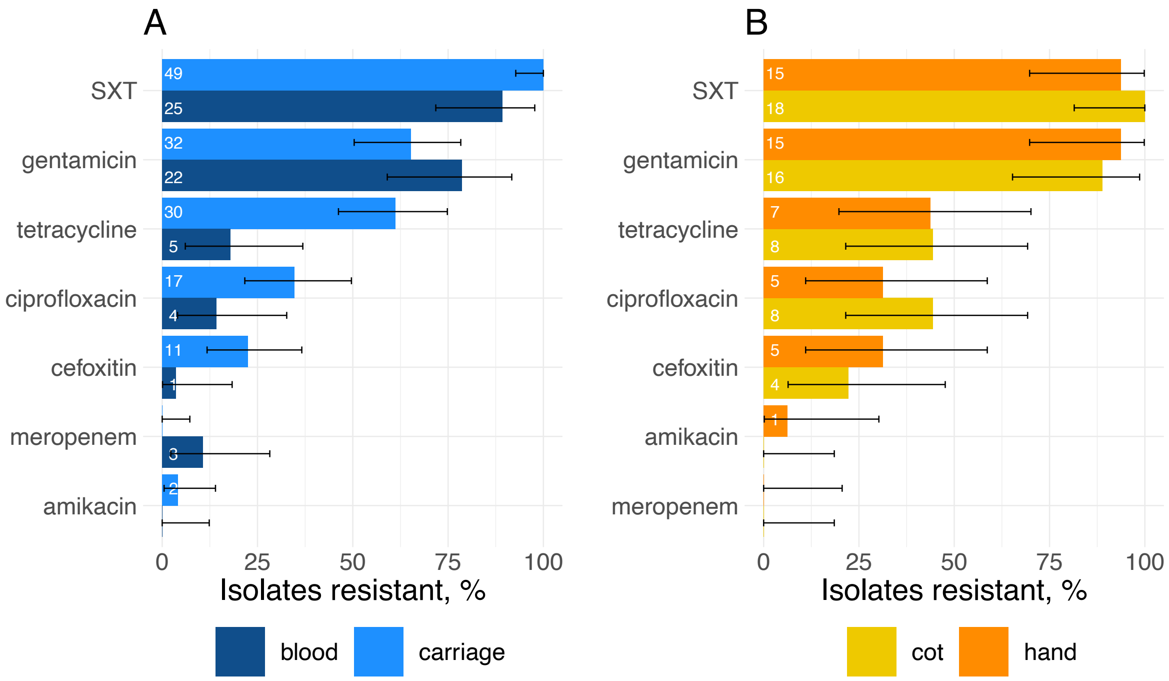

Figure S3 Frequencies of antimicrobial resistances in 3GC-R *K. pneumoniae* isolates A) in blood (dark blue) and carriage (light blue) and **B)** cot (yellow) and mothers' hands (orange).
